# Supplementary material for: Causal Relationship between Aging and Anorexia Nervosa: A White-Matter-Microstructure-Mediated Mendelian Randomization Analysis
Source: Biomedicines. 2024 Aug 16;12(8):1874. doi: 10.3390/biomedicines12081874 (PMC11351342; doi:10.3390/biomedicines12081874)
Supplement: Supplementary file 1 [file biomedicines-12-01874-s001.zip › Supplementary Figure/Figure S4/Singe SNP plot of MR analysis for TL to ubm-b-1898.pdf]

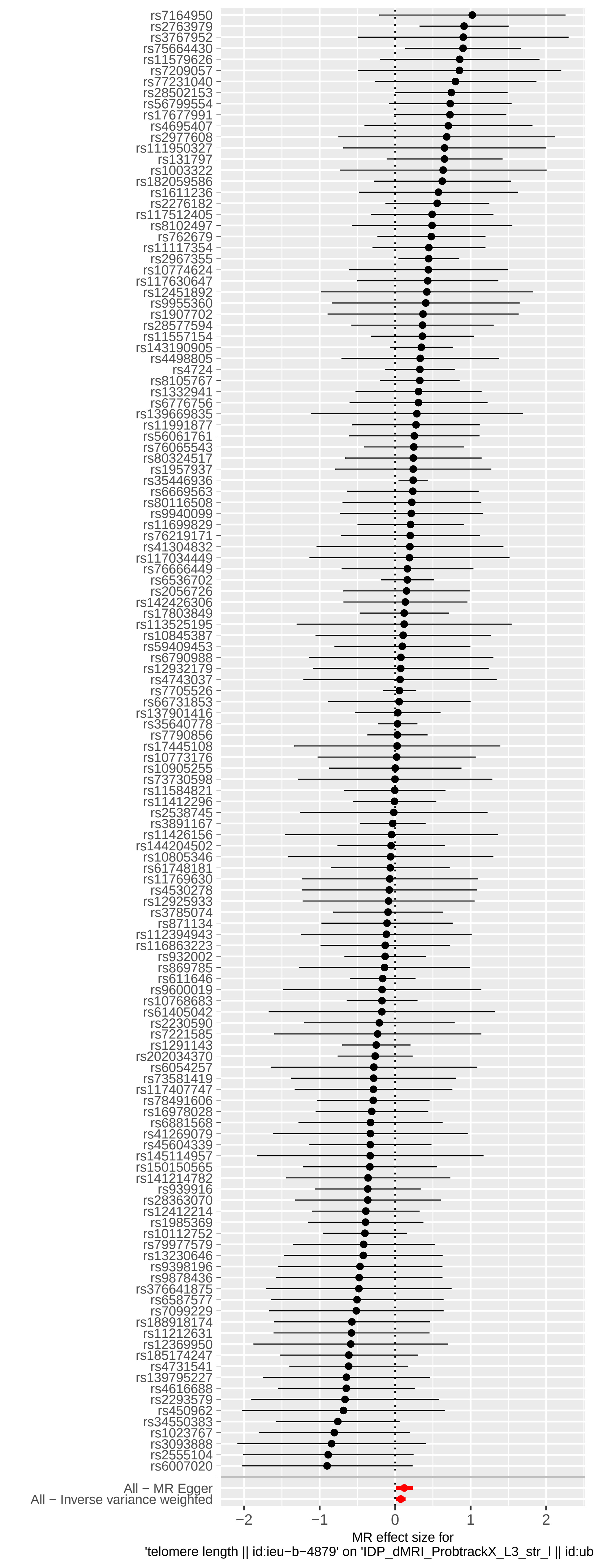
rs7164950
rs2763979
rs3767952
rs75664430
rs11579626
rs7209057
rs77231040
rs28502153
rs56799554
rs17677991
rs4695407
rs2977608
rs111950327
rs131797
rs1003322
rs182059586
rs1611236
rs2276182
rs117512405
rs8102497
rs762679
rs11117354
rs2967355
rs10774624
rs117630647
rs12451892
rs9955360
rs1907702
rs28577594
rs11557154
rs143190905
rs4498805
rs4724
rs8105767
rs1332941
rs6776756
rs139669835
rs11991877
rs56061761
rs76065543
rs80324517
rs1957937
rs35446936
rs6669563
rs80116508
rs9940099
rs11699829
rs76219171
rs41304832
rs117034449
rs76666449
rs6536702
rs2056726
rs142426306
rs17803849
rs113525195
rs10845387
rs59409453
rs6790988
rs12932179
rs4743037
rs7705526
rs66731853
rs137901416
rs35640778
rs7790856
rs17445108
rs10773176
rs10905255
rs73730598
rs11584821
rs11412296
rs2538745
rs3891167
rs11426156
rs144204502
rs10805346
rs61748181
rs11769630
rs4530278
rs12925933
rs3785074
rs871134
rs112394943
rs116863223
rs932002
rs869785
rs611646
rs9600019
rs10768683
rs61405042
rs2230590
rs7221585
rs1291143
rs202034370
rs6054257
rs73581419
rs117407747
rs78491606
rs16978028
rs6881568
rs41269079
rs45604339
rs145114957
rs150150565
rs141214782
rs939916
rs28363070
rs12412214
rs1985369
rs10112752
rs79977579
rs13230646
rs9398196
rs9878436
rs376641875
rs6587577
rs7099229
rs188918174
rs11212631
rs12369950
rs185174247
rs4731541
rs139795227
rs4616688
rs2293579
rs450962
rs34550383
rs1023767
rs3093888
rs2555104
rs6007020
All − MR Egger
All − Inverse variance weighted
−2
−1
0
1
2
MR effect size for
'telomere length || id:ieu−b−4879' on 'IDP_dMRI_ProbtrackX_L3_str_l || id:ub
